# The effectiveness of ultrasound bath and probe treatments on the quality of baking and shelf life of cupcakes

**DOI:** 10.1002/fsn3.1595

**Published:** 2020-05-16

**Authors:** Reza Esmaeilzadeh Kenari, Azita Nemati

**Affiliations:** ^1^ Department of Food Science and Technology Faculty of Agricultural Engineering Sari Agricultural Sciences and Natural Resources University Sari Iran

**Keywords:** baths, cupcakes, physicochemical properties, probes, shelf life, ultrasound waves

## Abstract

Uniform dispersion of emulsion particles of cake batter is very effective on the quality of the final product. A proper aeration process can produce a favorable spongy texture and desirable qualities in the product. Air containment and uniform distribution with smaller particles in the entire texture would be effective in achieving these goals. One of the newest physical methods to accomplish this matter is the use of ultrasound waves. The aim of this study was to investigate the effect of sounding the cake batter using two types of baths and ultrasound probes with two intensities (70% and 100%) over 0, 4, 6, and 8 min on the quality of cake baking and its shelf life. Porosity, cake texture and sensory acceptance, symmetry and uniformity indicator, water activity, and mold and yeast test for cake samples were taken during 14 days of storage in a factorial design. Examining the results showed that the probe ultrasound waves directly affected the quality of the cake, both bath and probe ultrasound types, which were applied for 6 min, improved the desired characteristics, while the increase in sounding time for up to 8 min decreased these parameters.

## Graphical abstract

The ultrasound improves baking characteristics and increases the shelf life of the cake, and the ultrasound effect of the probe is greater than the bath.

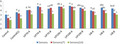

## 1 INTRODUCTION

Flour products are one of the commonly used food products around the world. Among the products, cakes are welcomed and well‐liked by consumers due to their organoleptic properties (Mastakidou, Blekas, & Paraskevopoulou, 2010). Cake is a special pastry with soft texture, which is considered as a calorie source placed between bread and biscuits. The cake's thin batter is an emulsion of oil in water, in which air bubbles are dispersed in a stable and uniform manner. In the mixing step of the cake batter, the air bubbles would be created in the cake batter structure, which will play a significant role in the final size of the product. One of the most important roles of oil used in cake formulation is to protect and maintain the air bubbles formed during mixing. Oils and fats play a very important role in creating porosity by maintaining air bubbles in the cake batter. The porosity created in the cake is due to the expansion of the air bubbles enclosed in the baking stage (Jafsri & Bhandar, 2007). Better distribution of fat and air bubbles in the batter structure leads to the cake's better final size and structure. The golden gel emulsifier is a white gel, which ingredients include monoglyceride and diglyceride, propylene glycol ester (PGE), glycerin, propylene glycol, potassium stearate, and water. The percentage and type of monoglycerides differ in the gel emulsion, which results in a slight difference in its functional properties. This gel emulsion is suitable for general use in all types of cakes, and the recommended dose is 0.5–1.5 percent based on the weight of the batter. Some of the functional properties of this compound include: (a) the expedited batter preparation and reduced mixing time; (b) the possibility of using a one‐step mixing method in the preparation of the cake batter; (c) increasing the oil and egg effect and its possible reduction in the formula; (d) high aeration and high batter strength; (e) the acceptance of air in the batter which increases the final product size; (f) creating a uniform surface and a soft and stable texture in the final product; (g) the improvement of the organoleptic properties of the cake; (h) the control of the staling; and (i) the improvement of the product appearance (Borneo, Aguirre, & Leon, 2010; Orcajo, Marcet, Paredes, & Diaz, 2013; Baixauli, Sanz, Salvador, & Fiszman, 2007).

Most of the high‐quality emulsifiers consumed in this type of product are largely imported, expensive, and costly, while some of them have chemical natures that can be problematic from the health and wellness point of view. Therefore, using high‐performance, modern methods can reduce the use of these additives. Ultrasonic waves are one of the new techniques for the preparation of emulsions using high pressure (Turabi, Sumnu, & Sahin, 2010).

The demand for new technologies that have less negative effects on the nutritional value of the products is increasing, because the quality of food is maintained better in nonthermal methods (Awad, Moharram, Shaltout, Askari, & Youssef, 2012). One of the newest physical methods in proper aerating is the use of ultrasound waves, which is a growing technology in various research fields that includes both the analysis and food refinement fields. Ultrasound is a form of energy produced by sound waves at a frequency much higher than the human hearing range. These waves have different categories based on the energy produced (wave power, wave intensity, wave density). Accordingly, ultrasound waves are placed in two groups of low energy (frequency higher than 100 KHz) and high energy (frequencies between 18 and 100 KHz) (Chemat, Huma, & Khan, 2011).

The main purpose of this research was applying ultrasound waves with high intensity to achieve a more uniform emulsion with smaller droplet size and more stability than conventional chemical methods and improve the quality of cupcakes.

In Tan, Chin, and Yusof (2011) explored the effect of ultrasound waves on cake. The results of the evaluations showed a decrease in the density of the batter and an increase in size and higher viscosity compared to the nonapplied samples, and the cake samples showed less hardness, which was more fluffy and desirable. Generally, ultrasound has a more specific effect on cake samples compared to batter samples.

Tan, Chin, Yusof, Taip, and Abdullah (2015) investigated the effect of ultrasound waves on a cake containing whey protein and without eggs. The results showed that in baked cake samples, sizes increased by 18%, and the density and hardness were, respectively, decreased by 18% and 65%. The results of the analysis showed that there was more air as small and stable bubbles in ultrasound‐applied samples.

Hokmabadi, Arianfar, and Sheikholeslami (2015) investigated the ultrasound effects on a cake containing triticale flour and tragacanth gum and examined the size, texture, porosity, and sensory properties of the sample with 0.6% gum and 20% triticale flour. The results showed that ultrasonic waves produced better aeration, so parameters such as size, porosity, and sensory properties were improved, the texture of the cake was softened, and the color of the cake was brightened inside and outside.

## 2 MATERIALS AND METHODS

### 2.1 Materials

Materials included flour, sugar, liquid vegetable oil, baking powder, and vanilla which were bought from a pastry supplier shop, and fresh eggs were provided one day before producing the sample and preserved in a refrigerator at 4°C. The emulgel, in the form of a golden gel, was prepared from Azarnoosh Shokoofeh Company.

### 2.2 Cake batter preparation

The compounds were weighed in accordance with the formulations as specified in Table 1. Oil, sugar powder, and egg were mixed with an electric mixer (128 rpm) for 3 min using the creaming method, and then, flour was added. The prepared batter was treated with an ultrasound bath and probed for 4, 6, and 8 min under intensities of 70% and 100%. The two control samples included the sample without ultrasound treatment and the sample without emulgel. Samples were placed in an oven for 20 min at 170°C for bottom surface and 120°C for upper surface(Fox, Smith, & Sahi, 2004; Tan et al., 2015).

**TABLE 1:** Recipe used for making cake batter

Ingredient	Concentration (g)
Flour	100.00
Sugar	115.00
Fat	60.00
Milk powder	70.00
Salt	2.50
Baking powder	4.00
Water	70.00
Liquid whole egg	80.00
Emulgel	6.00

### 2.3 Emulsification with ultrasound waves

The ultrasound waves were performed by an ultrasound machine for secondary homogenization of the prepared cake batter. The Elmasonic S (S30h) ultrasound bath machine made in Germany, with a constant power of 280 W and a constant frequency of 37 kHz, was used. The ultrasound process was applied indirectly to the cake batter over 4, 6, and 8 min.

For ultrasound probe treatment, the Ultrasonic Cell Disruptor (KS‐250F) made in China, with a constant frequency of 20 kHz and a power of 250 W and a voltage of 220 V, was used. The tip of the probe was placed in two‐thirds of the cake batter. During the homogenization, the sounding time was adjusted through the device at three time intervals (4, 6, and 8 min). In order to prevent the probe temperature from rising, the ultrasound was used discontinuously for different samples. Also, an ice bath was used to cool the sample and the probe. The sample was slowly shaken to ensure the quality of homogenization (Table 2).

**TABLE 2:** Process variables

Row	Ultrasound	Symbol	Amplitude	Time
1	Control	Control	–	–
2	Control	Control (1)	–	–
3	Probe	UP70‐4	70	4
4	Probe	UP70‐6	70	6
5	Probe	UP70‐8	70	8
6	Probe	UP100‐4	100	4
7	Probe	UP100‐6	100	6
8	Probe	UP100‐8	100	8
9	Bath	UB‐4	–	4
10	Bath	UB‐6	–	6
11	Bath	UB‐8	–	8

### 2.4 Water activity

The availability of water in a foodstuff is known as the water activity, which is in fact the ratio of the water vapor pressure in the foodstuff to pure water vapor pressure at the same temperature, which is useful for shelf life of the food. Water activity was measured after calibration of the apparatus (Novasina model: lab swift‐aw). Samples of cake crumb were placed in the device cell, and its water activity was read after 5 min exposure to a constant ambient temperature (Akesowan, 2009).

### 2.5 Cake texture

This test was performed using a texture analyzer (model: SANTAM STM‐5) device connected to a computer using the Texture Pro software. For this purpose, cubic parts with 40 '40' 40 'mm dimensions were first prepared for the whole sample, and then, their shells were removed. The samples were then subjected to a compression test under an aluminum cylindrical probe with 25 mm in diameter. The speed of the probe was 30 mm/min, and the compression (distance) amount was 30 mm during the test (Ronda, Gomes, Blanco, & Caballero, 2005). This test was also carried out after 1 and 7 days of maintaining the sample. The maximum force required to penetrate a cylindrical probe with a flat end (2 cm of diameter in 3.2 cm of height) at 30 mm/min from the center of the cake was calculated as a hardness indicator.

### 2.6 Sensory evaluation

The sensory evaluation was performed using the proposed method of Rajabzadeh (1991). For this purpose, the sensory characteristics of the cake in terms of form and shape, high‐level properties, low‐level properties, porosity, hardness and softness of the texture, chewiness, smell, and taste were evaluated by 10 panelists that were selected from trained people. The sensory characteristics of the cake were evaluated in terms of form and shape, upper surface properties, lower surface properties, emptiness and porosity, stiffness and softness, chewability, odor, flavor, and taste. The characteristics evaluation coefficient was from very bad (1) to very good (5). Finally, overall acceptance was calculated.

### 2.7 Porosity

To measure porosity, images were taken from inside of the cake samples. The taken images were given to Image J software, and by activating the ^8^bit part of the software, the so‐called grayscale images 4 were created, and then, the images were converted to binary images by activating the binary part of the software. Binary images are composed of a series of bright and dark points that calculate the ratio of bright points to dark points as an indicator of the porosity of the samples. Obviously, the higher ratio means that the amount of cavities in the cake structure is more or, in other words, its porosity is higher. In practice, by activating the Analysis section of the software, this ratio was calculated, and as a result, the porosity percentage of the samples was measured. This test was also carried out 24 hr after baking (Pardo, Ortiz‐Moreno, Mora‐Escobedo, Chanona‐Perez, & Necoechea‐Mondragon, 2008; Turabi et al., 2010; Wilderjans, Pareyt, Goesaert, Brijs, & Delcour, 2008).

### 2.8 Oven spring

Oven spring was determined by increasing the height of the batter after baking. To calculate this property, the difference in the height of the batter that was noted in the samples and the cake was determined immediately after baking (Shittu, Dixon, Awonorin, Sanni, & Maziya‐Dixon, 2008).

### 2.9 Symmetry and uniformity indicator

**FIGURE 1:**
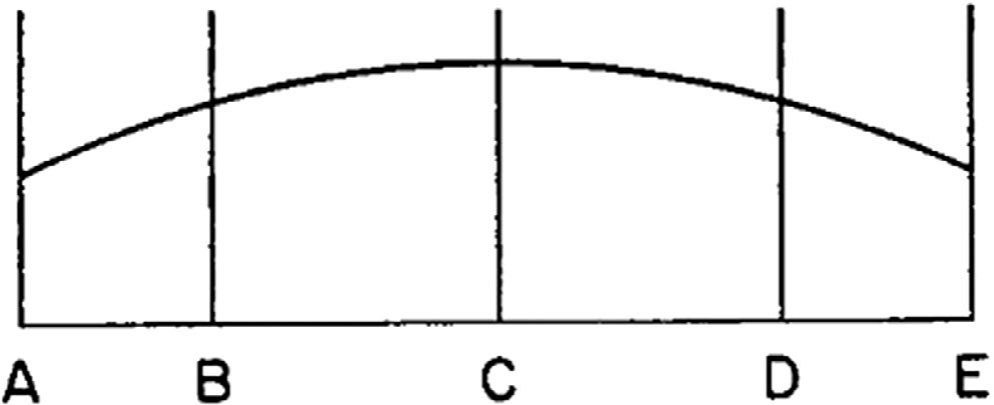
A schematic illustration of a transverse slice of a cake where C is the height at the center of the cake and B and D are the heights at the edges of the cake 2C‐B‐D = symmetry

The symmetry indicator was measured according to AACC 10‐91 (2000) method. The symmetry indicator is measurable based on the dimensions of the cake. For this purpose, the cake was cut into two halves and a pattern was used to measure the height of different points in the width section of the cake. Then, the symmetry indicator was determined by the AACC pattern method, which is based on the sum of heights in different points of the cake (Figure 1).

### 2.10 Evaluation of mold and yeast

In accordance with National Iranian Standard No. 10899, a YGC agar growth medium and pour plate method were used at dilution of 0.1 and 0.01.

### 2.11 Determining the amount of staling

Determining the amount of staling is the most important indicator in flour products and the main reason for the decrease in quality and nonacceptability during the shelf life of this product. This indicator has a direct relation with the product combinations. The standard method of AACC‐30‐74 sensory method was used to determine the amount of stale in cake samples. The samples were kept in plastic bags individually at room temperature and were given to panelists for sensory evaluation, and then, the cake samples were scored based on the relevant form. Score range for this trait was 1–5. Higher scores showed a lower staling rate than the control treatment. The sensory characteristics of the cake were evaluated in terms of stiffness and softness, chewability, and taste. In addition, the texture analyzer was used to evaluate the texture or staling of cake samples.

## 3 STATISTICAL DESIGN

A factorial design and Duncan test at 95% confidence level were used for statistical analysis of the data. In order to reduce the error, all tests would be performed in 3 repetitions. The software used for the analysis was SPSS v.20. Excel 2007 was used to draw different shapes and curves from the mean of the data.

## 4 RESULTS AND DISCUSSION

### 4.1 Texture

**FIGURE 2:**
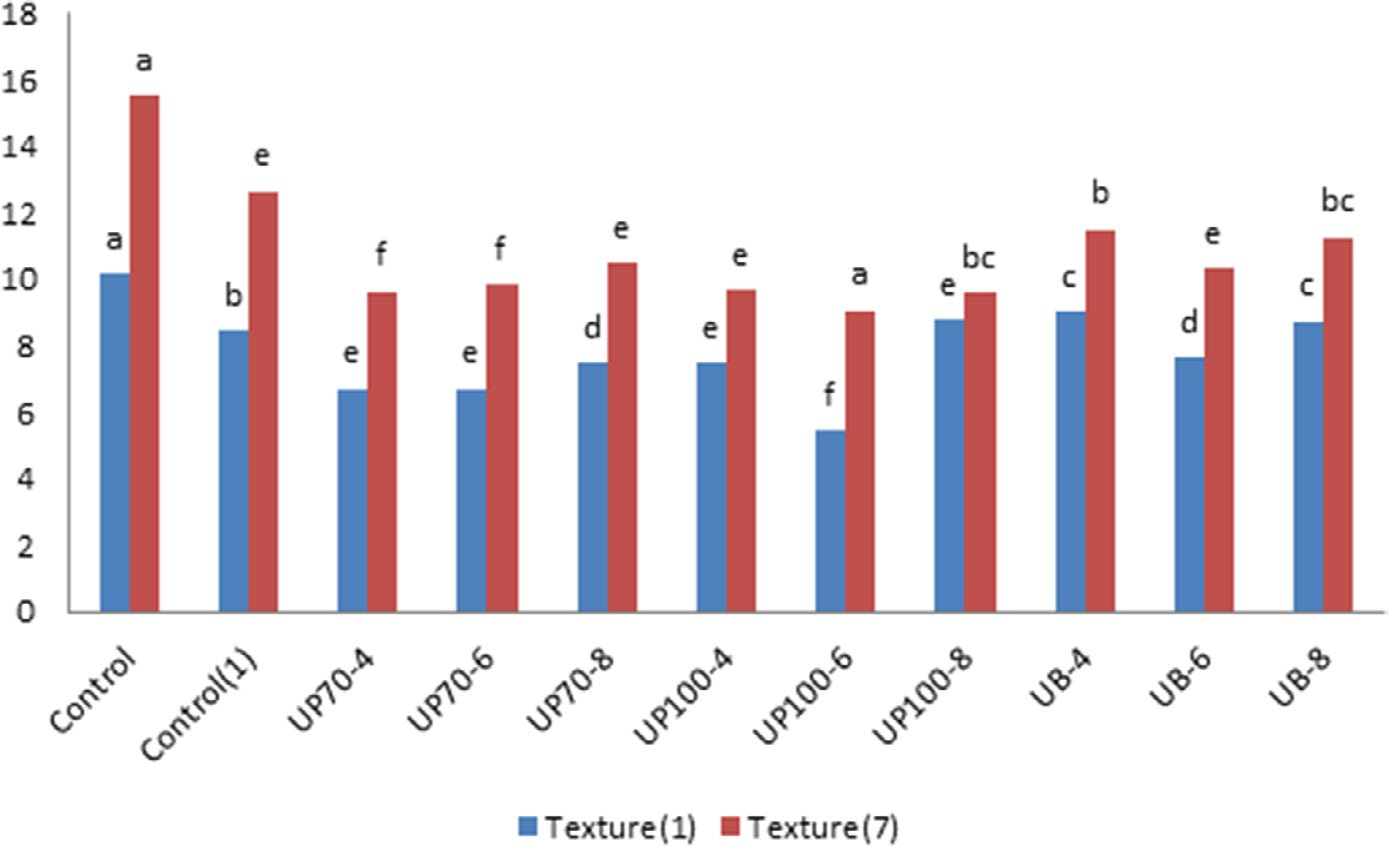
Effect of time and intensity of ultrasound on cake texture. (Control) Sample without ultrasound and emulgel. (Control1) Sample without ultrasound. (UP70‐4) Sample pretreated with probe ultrasound in intensity of 70% for 4 min. (UP70‐6) Sample pretreated with probe ultrasound in intensity of 70% for 6 min. (UP70‐8) Sample pretreated with probe ultrasound in intensity of 70% for 8 min. (UP100‐4) Sample pretreated with probe ultrasound in intensity of 100% for4 min. (UP100‐6) Sample pretreated with probe ultrasound in intensity of 100% for 6 min. (UB‐4) Sample pretreated with bath ultrasound for 4 min. (UB‐6) Sample pretreated with bath ultrasound for 6 min. (UB‐8) Sample pretreated with bath ultrasound for 8 min

In Figure 2, the effect of ultrasound wave intensity and the process time on the cake texture is observed. The independent effect of ultrasound time was significant at 1%, and the interactional effect between ultrasound time and intensity was also significant at this level. At a time interval of one day after baking, ultrasound probe samples with 100% intensity for 6 min had less hardness than the other samples. Texture hardness is associated with several factors such as humidity level, porosity in texture, and size. Effective aeration can improve the amount of the texture porosity. Due to the sounding process, the hardness of the texture is reduced, which can be attributed to better aeration and uniform distribution of gas cells in the final product.

By increasing the storage time, the texture hardness was also increased, which is natural due to the staling phenomenon and the removal of moisture from the inside of the cake. This is because of the ability of ultrasound and emulgel to keep moisture and prevent the bacteria growth, which results in texture softness and complete postponement of the hardness process. Ultrasound‐treated cakes were softer than the control samples and retained their soft texture throughout the entire shelf life. The samples with emulgel showed softer textures than the nongel samples. Sheikholeslami, Mortazavi, Pourazarang, and Nasiri (2010) stated that the sounding process reduced the hardness of the bread texture due to the increase in the number of cavities due to better aeration and the uniform distribution of gas cells in the batter texture and the final product, which was in line with the present study. In (2018) Naghipour. indicated that one of the factors of moisture preservation in foodstuffs is the presence of higher levels and smaller particles, in which the water is enclosed and loses the ability to get out of the texture of the final product during the baking process and afterward. Such an effect would not be seen during the longer sounding process because of the destructive effects of these waves on proteins and internal texture (Table 3).

**TABLE 3:** Effect of process variables on texture, porosity and oven spring of cupcake after baking

Treatment	Oven spring	Texture (1)	Texture (7)	Porosity
Control	1.733 ± 0.11^f^	10.16 ± 0.08^a^	15.54 ± 0.12^a^	19.92 ± 0.43^f^
Control (1)	1.833 ± 0.05^f^	8.47 ± 0.04^b^	12.65 ± 0.19^e^	21.95 ± 0.87^ef^
UP70‐4	2.266 ± 0.11^e^	6.72 ± 0.05^e^	9.64 ± 0.08^f^	31.06 ± 0.76^c^
UP70‐6	2.700 ± 0.11^cd^	6.72 ± 0.05^e^	9.83 ± 0.26^f^	34.87 ± 0.88^b^
UP70‐8	2.633 ± 0.26^d^	7.50 ± 0.04^d^	10.48 ± 0.1^e^	21.18 ± 1.27^f^
UP100‐4	2.600 ± 0.20^d^	7.46 ± 0.04^e^	9.66 ± 0.3^e^	31.34 ± 0.74^c^
UP100‐6	3.333 ± 0.30^a^	5.48 ± 0.09^f^	9.08 ± 0.09^a^	42.39 ± 1.98^a^
UP100‐8	3.133 ± 0.11^ab^	8.79 ± 0.11^e^	9.62 ± 0.23^bc^	28.62 ± 1.20^cd^
UB‐4	2.233 ± 0.05^e^	9.04 ± 0.15^c^	11.51 ± 0.06^b^	24.73 ± 2.7^e^
UB‐6	2.666 ± 0.15^d^	7.62 ± 0.16^d^	10.31 ± 0.2^e^	27.58 ± 3.17^d^
UB‐8	2.666 ± 0.11^d^	8.75 ± 0.09^c^	11.26 ± 0.24^bc^	20.12 ± 1.47^f^

### 4.2 Oven spring

**FIGURE 3:**
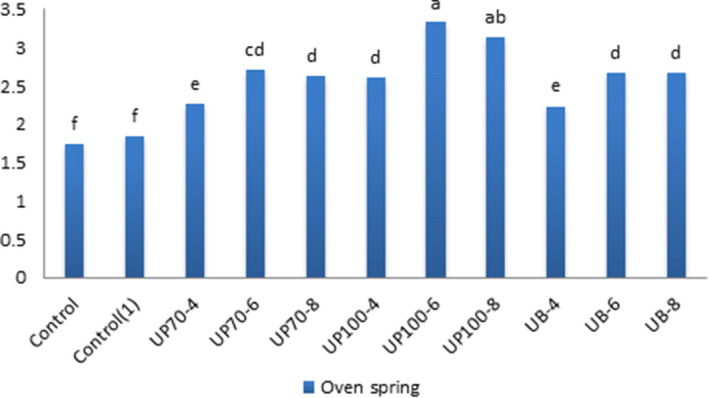
Effect of time and intensity of ultrasound on the height of the cake after baking. (Control) Sample without ultrasound and emulgel. (Control1) Sample without ultrasound. (UP70‐4) Sample pretreated with probe ultrasound in intensity of 70% for 4 min. (UP70‐6) Sample pretreated with probe ultrasound in intensity of 70% for 6 min. (UP70‐8) Sample pretreated with probe ultrasound in intensity of 70% for 8 min. (UP100‐4) Sample pretreated with probe ultrasound in intensity of 100% for 4 min. (UP100‐6) Sample pretreated with probe ultrasound in intensity of 100% for 6 min. (UB‐4) Sample pretreated with bath ultrasound for 4 min. (UB‐6) Sample pretreated with bath ultrasound for6 min. (UB‐8) Sample pretreated with bath ultrasound for 8 min

Figure 3 shows the effect of time and intensity of ultrasound on the height of the cake after baking. The independent effect of ultrasound was significant at 1% level, and the interaction effect between ultrasound time and intensity was also significant at this level. Both bath and probe ultrasound types, which were applied for 6 min, showed higher levels of height increase after baking. The height differences in sample without emulgel and without ultrasound treatment were negligible.

The ability of the batter to keep air bubbles intact in the ultrasound application process, that leads to increased aeration of the batter, is one of the effective factors in improving the shape and size properties of the cake. In addition to mechanical aeration in the mixing process, chemical aeration is also effective during the baking process in creating the final size of the product. Improving the rheological properties of the batter and creating interphase surfaces by emulsifier is effective in stabilizing the foam during baking. Hokmabadi et al. (2015), Sheikholeslami et al. (2010), Turabi et al. (2010), and Ashwini, Jyotsna, and Indrani (2009) reported that emulsifiers and ultrasonic waves, by reducing the surface tension of the fat phase, improve its ability to spread in the cake batter, which will increase the strength of the cake and its softness (Figure 3).

### 4.3 Sensory evaluation

**FIGURE 4:**
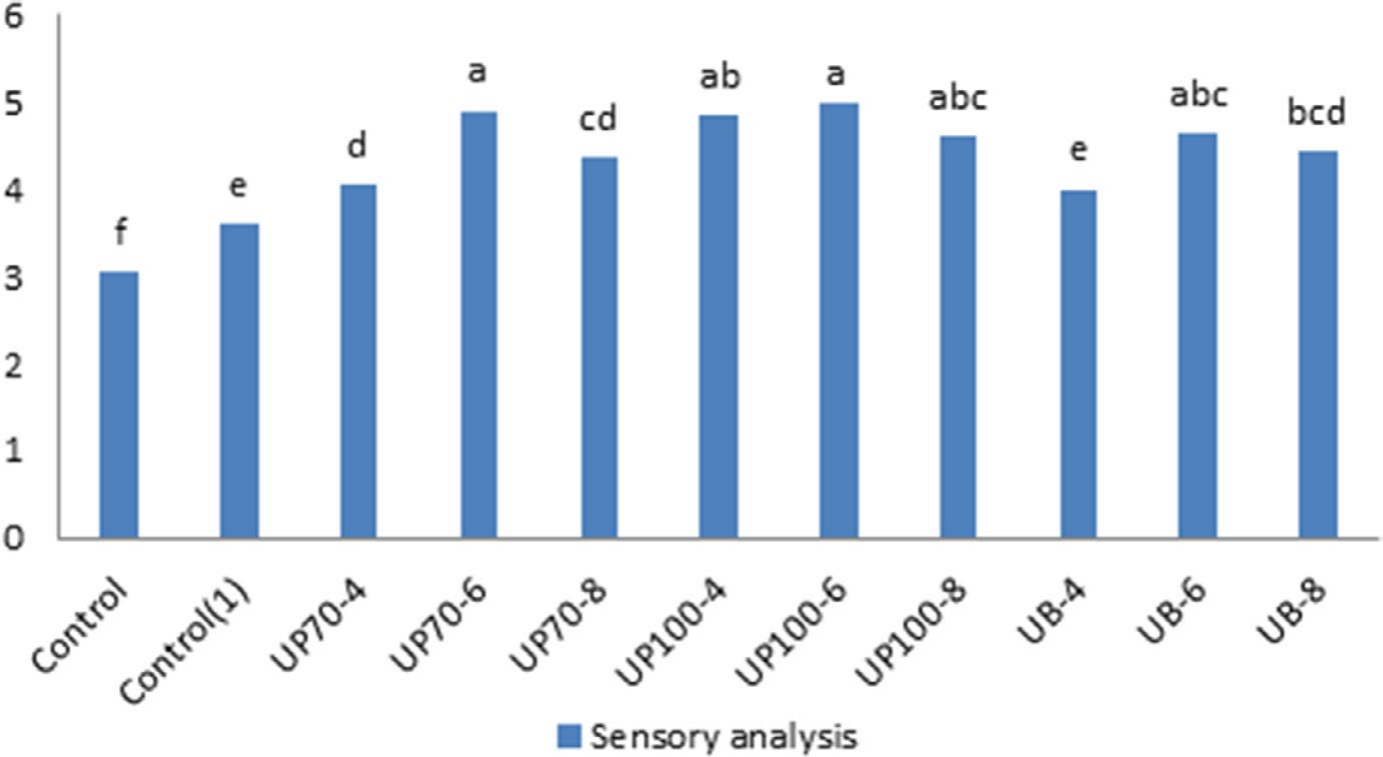
Effect of time and intensity of ultrasound on sensory evaluation of cake. (Control) Sample without ultrasound and emulgel. (Control1) Sample without ultrasound. (UP70‐4) Sample pretreated with probe ultrasound in intensity of 70% for 4 min. (UP70‐6) Sample pretreated with probe ultrasound in intensity of 70% for 6 min. (UP70‐8) Sample pretreated with probe ultrasound in intensity of 70% for 8 min. (UP100‐4) Sample pretreated with probe ultrasound in intensity of 100% for 4 min. (UP100‐6) Sample pretreated with probe ultrasound in intensity of 100% for 6 min. (UB‐4) Sample pretreated with bath ultrasound for 4 min. (UB‐6) Sample pretreated with bath ultrasound for 6 min. (UB‐8) Sample pretreated with bath ultrasound for 8 min

The independent effect of ultrasound time was significant at 1% level, but the interaction effect between ultrasound time and intensity was not significant at this level. The higher overall acceptance was due to better texture, more proper color, as well as better oral sensation, increased moisture, and porosity of the product. There was a high correlation between the texture and the understanding of the sensory properties. Maintaining more moisture due to the presence of smaller particles and the creation of soft texture in the product and samples with more porosity will make the product more desirable. Texture is an important qualitative feature that greatly affects the consumer's sensory experience of a product. The number, size, and distribution of the air cells affect the texture and therefore the quality of the product. The number and size of the air cells affect the structure of the cake, the thickness of the cell walls, and consequently the force required to bite, chew, and swallow it. Samples treated with ultrasound probes got higher scores compared to the bath type, and the samples without emulgel and without ultrasound treatment had the lowest scores. Naghipour et al. (2018) obtained higher scores from tasting panelists because of preserving the moisture and characteristics of the cake shell and the presence of smaller particles by the cavitation phenomena that had the slightest change in the baking process (Figure 4; Tables 4 and 5).

**TABLE 4:** Effect of process variables on sensory evaluation and stale ratio in cupcakes

Treatment	Sensory	Sensory (7)	Sensory (14)
Control	3.06 ± 0.11^f^	2.33 ± 0.28^e^	1.86 ± 0.23^e^
Control (1)	3.60 ± 0.10^e^	3.33 ± 0.30^d^	2.23 ± 0.25^e^
UP70‐4	4.06 ± 0.11^d^	4.10 ± 0.36^bc^	3.13 ± 0.23^c^
UP70‐6	4.9 ± 0.11^a^	4.73 ± 0.46^a^	3.33 ± 0.30^c^
UP70‐8	4.36 ± 0.32^cd^	4.56 ± 0.40^ab^	3.23 ± 0.25^c^
UP100‐4	4.86 ± 0.23^ab^	4.80 ± 0.20^a^	4.43 ± 0.20^a^
UP100‐6	5 ± 0^a^	4.83 ± 0.28^a^	4.06 ± 0.11^ab^
UP100‐8	4.60 ± 0.40^abc^	4.36 ± 0.20^abc^	3.93 ± 0.11^b^
UB‐4	4 ± 0.2^de^	3.80 ± 0.20^cd^	3.00 ± 0.20^c^
UB‐6	4.63 ± 0.23^abc^	4.06 ± 0.11^bc^	3.43 ± 0.4^c^
UB‐8	4.43 ± 0.40^bcd^	3.43 ± 0.40^d^	3.06 ± 0.11^c^

**TABLE 5:** Results of analysis of variance (degree of freedom and mean square) for sensory evaluation, staling, and cupcake texture

Source	*df*	MS				
		Sensory	Sensory (7)	Sensory (14)	Texture	Texture (1)
Model	10	1.075**	1.774**	1.690**	10.458**	5.200**
Amp	2	0.504**	1.840**	1.858**	8.033**	5.216**
Time	2	0.708**	0.431*	0.090 ns	1.233**	7.047**
Time * Amp	4	0.156 ns	0.199 ns	0.152*	0.434**	1.807**
Error	22	0.057**	0.097**	0.056**	0.037**	0.091**
Total	33	–	–	–	–	–

### 4.4 Staling

Staling is the most important indicator in flour products, and it is the main cause of quality deterioration and nonacceptability during the storage time of this product. This indicator has a direct relationship with the product combinations. By increasing the storage time, the staling significantly (*p* < .01) increased in all of the samples tested, but the intensity of staling was similar in all the treatments. The lowest sensory score in the staling test was in the second week of storage for a sample without ultrasound and emulgel. The ability of the batter to keep the gas and moisture would preserve the quality of the cake during the storage time and delay the staling process.

**FIGURE 5:**
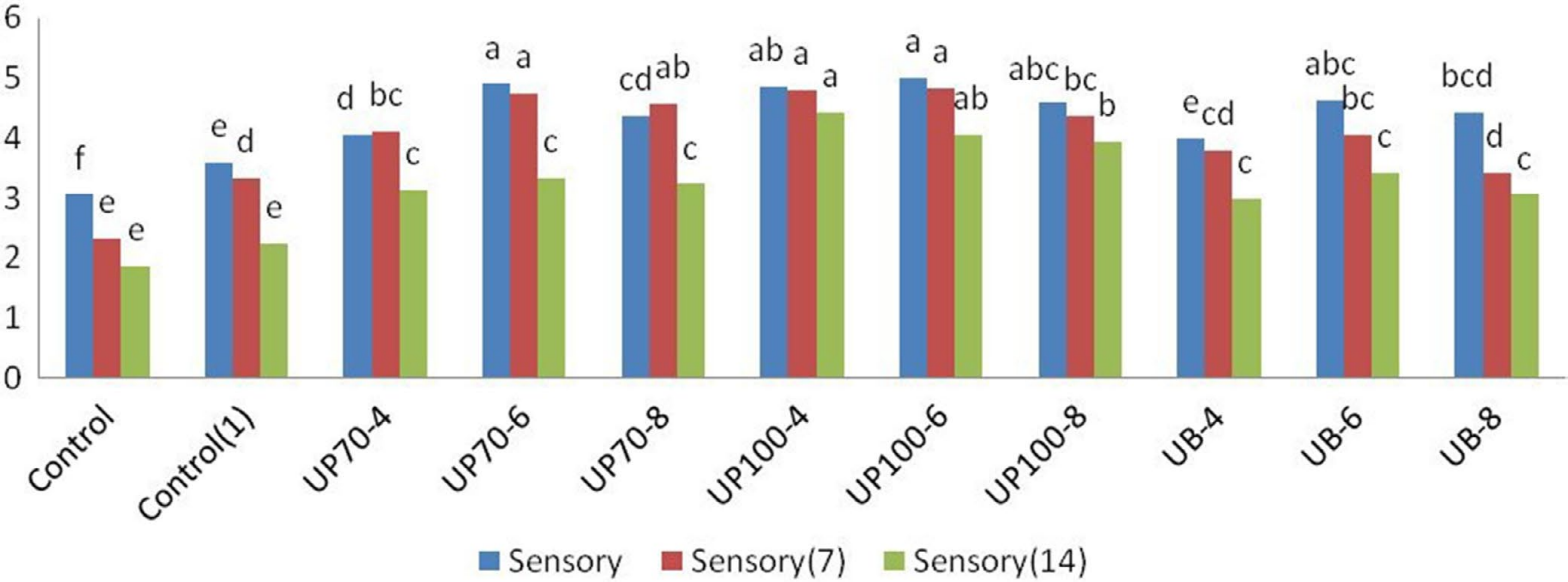
Effect of time and intensity of ultrasound on the process of cake staling. (Control) Sample without ultrasound and emulgel. (Control1) Sample without ultrasound. (UP70‐4) Sample pretreated with probe ultrasound in intensity of 70% for 4 min. (UP70‐6) Sample pretreated with probe ultrasound in intensity of 70% for 6 min. (UP70‐8) Sample pretreated with probe ultrasound in intensity of 70% for 8 min. (UP100‐4) Sample pretreated with probe ultrasound in intensity of 100% for 4 min. (UP100‐6) Sample pretreated with probe ultrasound in intensity of 100% for 6 min. (UB‐4) Sample pretreated with bath ultrasound for 4 min. (UB‐6) Sample pretreated with bath ultrasound for 6 min. (UB‐8) Sample pretreated with bath ultrasound for 8 min

Factors such as amylopectin retrogradation, amorphous polymorphic rearrangement, decreased moisture, or moisture distribution between the amorphous and crystalline regions are involved in texture stale and hardness (Naghipour 2018). One of the factors of keeping moisture in the food texture is the presence of higher levels of small particles in them, in which water is enclosed and would not be able to get out of the texture of the final product. This would not happen over the longer application of ultrasound because of its destructive effects on the internal texture. In Turabi et al. (2010) published a report on the role of monoglyceride as a softener and antistale agent in the cake (Figure 5).

### 4.5 Symmetry and uniformity

The independent effect of ultrasound time was significant at 1% level, and the interaction effect between ultrasound time and intensity on the symmetry and uniformity of cake was significant at 5% level. Samples treated with ultrasound probes got higher values compared to the bath type. The increase in sounding time for up to 8 min decreased these values.

**FIGURE 6:**
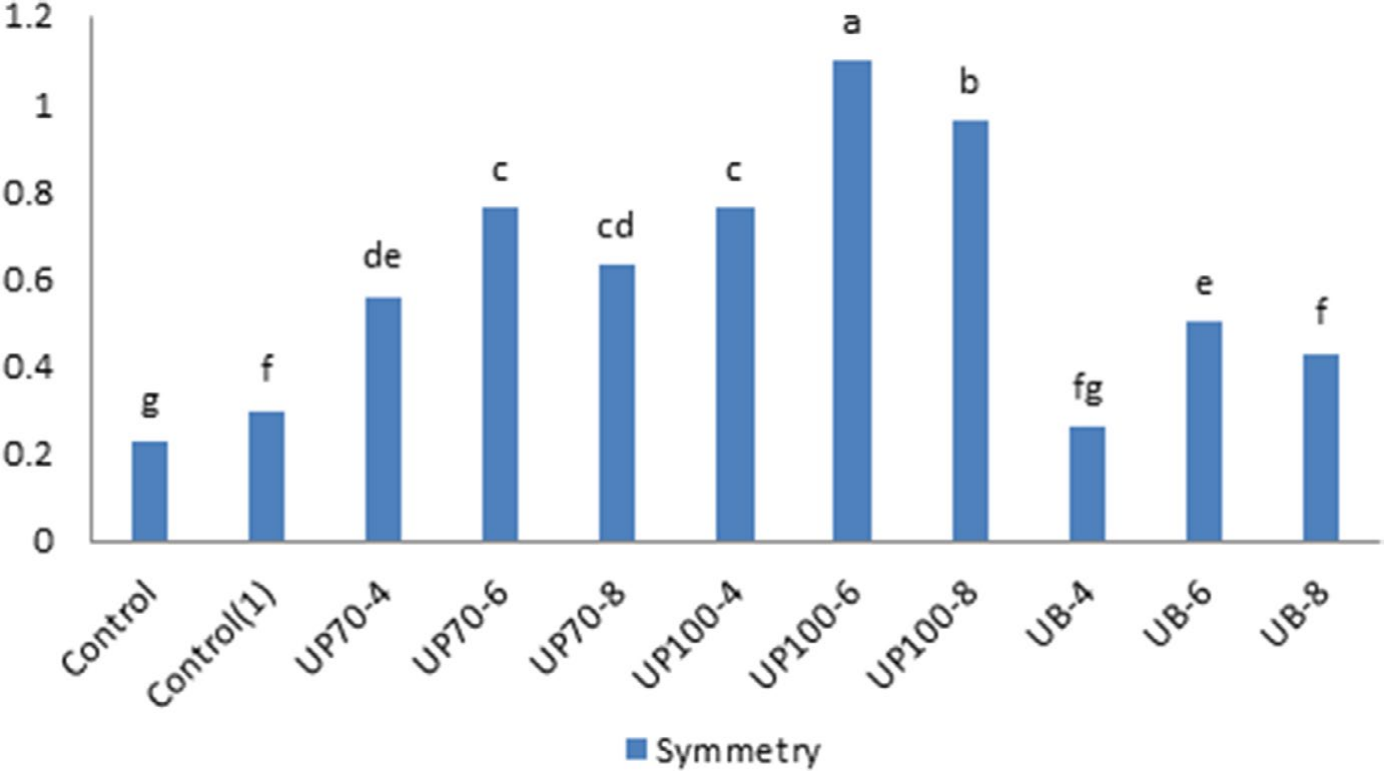
Effect of time and intensity of ultrasound on the symmetry and uniformity of the cake. (Control) Sample without ultrasound and emulgel. (Control1) Sample without ultrasound. (UP70‐4) Sample pretreated with probe ultrasound in intensity of 70% for 4 min. (UP70‐6) Sample pretreated with probe ultrasound in intensity of 70% for 6 min. (UP70‐8) Sample pretreated with probe ultrasound in intensity of 70% for 8 min. (UP100‐4) Sample pretreated with probe ultrasound in intensity of 100% for 4 min. (UP100‐6) Sample pretreated with probe ultrasound in intensity of 100% for 6 min. (UB‐4) Sample pretreated with bath ultrasound for 4 min. (UB‐6) Sample pretreated with bath ultrasound for 6 min. (UB‐8) Sample pretreated with bath ultrasound for 8 min

The ability of the batter to maintain air bubbles during the application of ultrasound, which leads to increased aeration of the batter, is one of the effective factors of improving the shape and size properties of the cake (Figure 6).

### 4.6 Water activity

**FIGURE 7:**
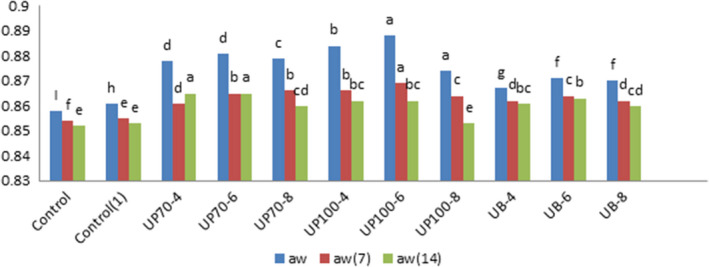
Effect of time and intensity of ultrasound on water activity of cake. (Control) Sample without ultrasound and emulgel. (Control1) Sample without ultrasound. (UP70‐4) Sample pretreated with probe ultrasound in intensity of 70% for 4 min. (UP70‐6) Sample pretreated with probe ultrasound in intensity of 70% for 6 min. (UP70‐8) Sample pretreated with probe ultrasound in intensity of 70% for 8 min. (UP100‐4) Sample pretreated with probe ultrasound in intensity of 100% for4 min. (UP100‐6) Sample pretreated with probe ultrasound in intensity of 100% for 6 min. (UB‐4) Sample pretreated with bath ultrasound for 4 min. (UB‐6) Sample pretreated with bath ultrasound for 6 min. (UB‐8) Sample pretreated with bath ultrasound for 8 min

A desirable aeration process can produce proper spongy texture and control the moisture content of the final product. Air retention and uniform distribution with smaller particles in the entire texture will be effective in achieving these goals. According to Figure 7, the water activity of the samples decreased during the storage time. The independent effect of ultrasound time and the interaction effect between ultrasound time and intensity on the water activity were significant at 1% level. Samples treated with ultrasound probes got higher values compared to the bath type. However, at the end of the storage time, the time and intensity of ultrasound showed no significant effect on water activity (Tables 6 and 7).

**TABLE 6:** Effect of process variables on water activity, symmetry and uniformity of cupcakes

Treatment	*a*_w_	*a*_w_ (7)	*a*_w_ (14)	Symmetry
Control	0.858 ± 0.001^I^	0.854 ± 0.057^f^	0.852 ± 0.005^e^	0.23 ± 0.057^g^
Control (1)	0.861 ± 0.001^h^	0.855 ± 0.005^e^	0.853 ± 0.001^e^	0.30 ± 0.10^fg^
UP70‐4	0.878 ± 0.005^d^	0.861 ± 0.005^d^	0.865 ± 0.001^a^	0.56 ± 0.05^de^
UP70‐6	0.881 ± 0.005^d^	0.865 ± 0.005^b^	0.865 ± 0.005^a^	0.76 ± 0.11^c^
UP70‐8	0.879 ± 0.005^c^	0.866 ± 0.015^b^	0.860 ± 0.005^cd^	0.63 ± 0.11^cd^
UP100‐4	0.884 ± 0.005^b^	0.866 ± 0.005^b^	0.862 ± 0.001^bc^	0.76 ± 0.15^c^
UP100‐6	0.888 ± 0.01^a^	0.869 ± 0.005^a^	0.862 ± 0.001^bc^	1.1 ± 0.11^a^
UP100‐8	0.874 ± 0.01^e^	0.864 ± 0.005^c^	0.853 ± 0.001^e^	0.96 ± 0.05^b^
UB‐4	0.867 ± 0.005^g^	0.862 ± 0.005^d^	0.863 ± 0.005^bc^	0.26 ± 0.11^fg^
UB‐6	0.871 ± 0.01^f^	0.864 ± 0.005^c^	0.861 ± 0.001^b^	0.5 ± 0.10^de^
UB‐8	0.870 ± 0.005^f^	0.862 ± 0.005^d^	0.860 ± 0.001^cd^	0.43 ± 0.05^ef^

**TABLE 7:** Results of the analysis of variance (degrees of freedom and mean square) for water activity, porosity, cake height after baking, symmetry and uniformity in cupcakes

Source	*df*	MS					
		*a*_w_	*a*_w_ (7)	*a*_w_ (14)	Oven spring	Symmetry	Porosity
Model	10	0**	61,650**	22,055 ns	0.76**	0.266**	55.138**
Amp	2	0**	21,360**	17,969 ns	0.412**	0.485**	56.211**
Time	2	47,810**	22,700**	26,955 ns	0.871**	0.134**	113.351**
Time * Amp	4	67,260**	11,200**	26,956 ns	0.065**	0.030*	8.672**
Error	22	78,790**	51,520**	22,056 ns	0.027 ns	0.010**	–
Total	33	–	–	–	–	–	–

Note

ns: not meaningful at 1% and 5%.

* Significant at 5% level.

** Significant at 1%

### 4.7 Porosity

**FIGURE 8:**
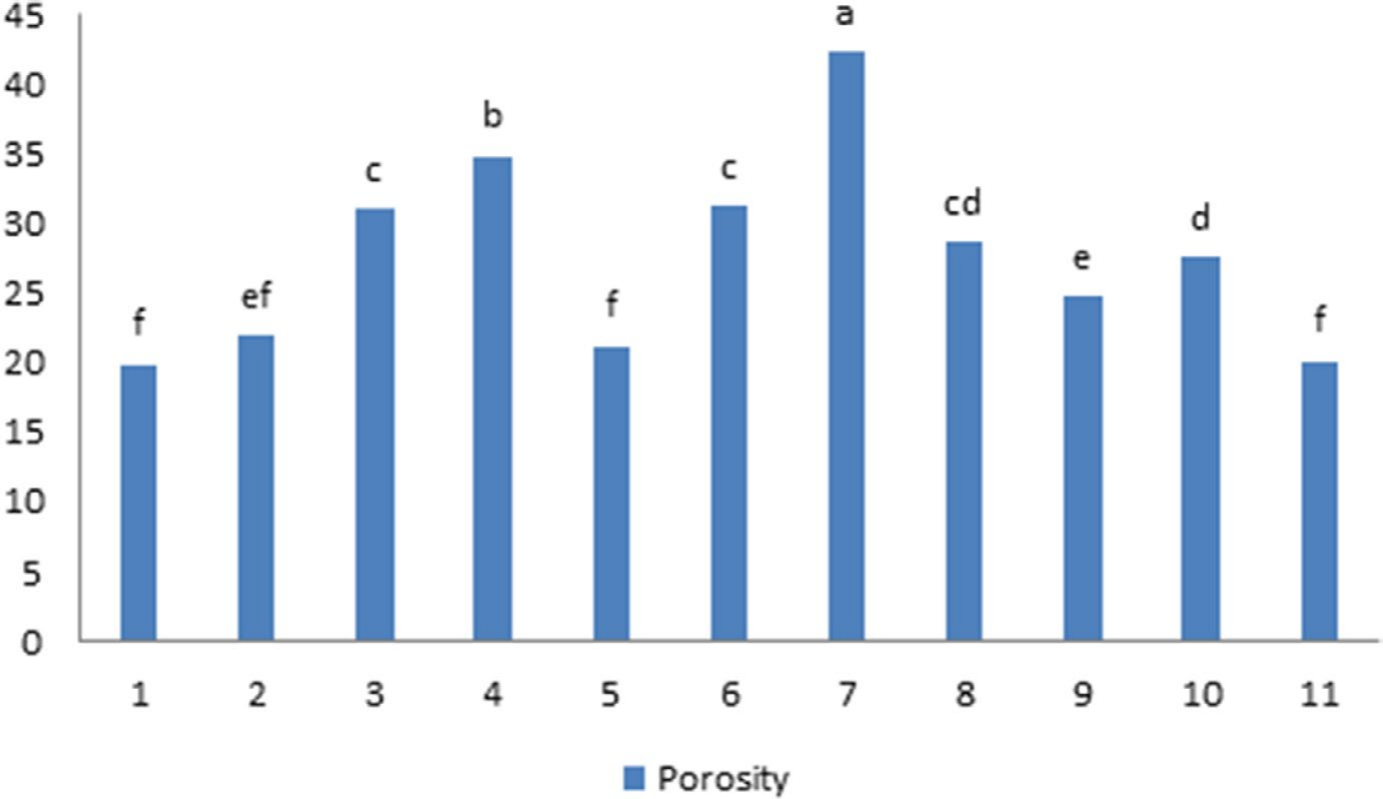
Effect of time and intensity of ultrasound on porosity of cake texture (Control) Sample without ultrasound and emulgel. (Control1) Sample without ultrasound. (UP70‐4). Sample pretreated with probe ultrasound in intensity of 70% for 4 min. (UP70‐6) Sample pretreated with probe ultrasound in intensity of 70% for 6 min. (UP70‐8) Sample pretreated with probe ultrasound in intensity of 70% for 8 min. (UP100‐4) Sample pretreated with probe ultrasound in intensity of 100% for 4 min. (UP100‐6) Sample pretreated with probe ultrasound in intensity of 100% for 6 min.(UB‐4) Sample pretreated with bath ultrasound for 4 min. (UB‐6) Sample pretreated with bath ultrasound for 6 min. (UB‐8) Sample pretreated with bath ultrasound for 8 min

According to Figure 8, the most porous structure is observed in ultrasound probe sample with 100% intensity for 6 min. Increasing the time of ultrasound application for up to 8 min reduced the values of porosity. The independent effect of ultrasound time and the interaction effect between ultrasound time and intensity were significant for cake porosity at 1%. The direct application of ultrasound has a greater effect on the porosity of the samples. The use of ultrasound waves over longer periods of time reduced porosity, which could be due to the greater effect of these waves on proteins. Another important factor in the formation of bubbles and its stability in the system are water‐soluble proteins. On the one hand, proteins, as surface active agents, reduce the surface tension between fluid and air, and on the other hand, they cause bubble stability through the adhesive film in the midpoint. Aeration with ultrasonic waves creates a texture with multiple, uniform, and completely porous cavities in the final product. Naghipour et al. (2018) made a similar report, including the advantages of ultrasound aeration during emulsion production, the creation of texture with multiple, uniform, and completely porous cavities in the final product.

### 4.8 Growth of mold and yeast in the cake

The most important factors affecting the growth of mold and yeast are the moisture of the product and the microbiological quality of the raw materials in the product. By the 14th day, all treatments showed no mold and yeast growth (Table 8).

**TABLE 8:** Growth of mold and yeast in the cake

Treatment	Mold			Yeast		
	Day			Day		
	1	7	14	1	7	14
Control	N	N	<10	N	N	<10
Control (1)	N	N	<10	N	N	<10
UP70‐4	N	N	<10	N	N	<10
UP70‐6	N	N	<10	N	N	<10
UP70‐8	N	N	<10	N	N	<10
UP100‐4	N	N	<10	N	N	<10
UP100‐6	N	N	<10	N	N	<10
UP100‐8	N	N	<10	N	N	<10
UB‐4	N	N	<10	N	N	<10
UB‐6	N	N	<10	N	N	<10
UB‐8	N	N	<10	N	N	<10

Abbreviation: N: negative.

## 5 CONCLUSION

The most important result in this study was the high performance of ultrasound waves in improving the emulsification of oil in water. Emulsification with ultrasonic waves compared to the conventional method of using the emulsifiers alone provides high‐quality emulsion and therefore provides suitable emulsifiers and foaming properties. Emulsification with ultrasonic waves makes the batter mixing faster and increases the quality of the batter and the texture of the cake. According to the results of sounding the batter, the porosity and the size of the cake were improved; therefore, the product was softer and the staling was delayed. Comparing the results of ultrasound bath and ultrasound probe, the probe type had more effect on the quality of the cake. Ultrasound waves with 100% intensity in 6 min resulted in more suitable outcomes. Increasing the time of ultrasound application for up to 8 min reduced the amount of these parameters.

## CONFLICT OF INTEREST

The authors declare no conflict of interest.

## ETHICAL APPROVAL

The human and animal testing was unnecessary in the current study.
